# Text Mining for Translational Bioinformatics

**DOI:** 10.1155/2015/368264

**Published:** 2015-08-25

**Authors:** Hong-Jie Dai, Chih-Hsuan Wei, Hung-Yu Kao, Rey-Long Liu, Richard Tzong-Han Tsai, Zhiyong Lu

**Affiliations:** ^1^Department of Computer Science and Information Engineering, National Taitung University, Taitung City 950, Taiwan; ^2^National Center for Biotechnology Information, National Library of Medicine, 8600 Rockville Pike, Bethesda, MD 20894, USA; ^3^Department of Computer Science and Information Engineering, National Cheng Kung University, Tainan 701, Taiwan; ^4^Department of Medical Informatics, Tzu Chi University, Hualien 970, Taiwan; ^5^Department of Computer Science and Information Engineering, National Central University, Taoyuan 320, Taiwan

Translational bioinformatics is an emerging field with a fascinating aim to develop novel computational techniques to facilitate traditional translational research through the convergence of molecular bioinformatics, biostatistics, statistical genetics, and clinical informatics. Translational bioinformatics is now more powerful than ever and has become a clinch between biological findings and clinical informatics. The computational techniques contribute by integrating multidimensional data consisting of medications, diseases, and genomes with clinical and pathological features. They are applied in various aspects with the hope of uncovering therapeutic targets and biomarkers of patient response. However, the accumulation of rich data from past studies, advancement of new experimental techniques, and ease of access to publications nowadays result in enormous repositories of scientific literatures and biomedical data, hindering the translation of molecular understandings into technologies that could impact patients. Text mining is an established field, but its application for translational bioinformatics is still a novel research direction with enormous research potential. The present issue emphasizes the application of text mining on biomedical/clinical publications and knowledge bases to facilitate the discovery and management of translational medical research knowledge.

Rapid growth of disease related biomedical literature makes the traditional information retrieval techniques insufficient to fulfill searchers' information needs. In the paper “Disease Related Knowledge Summarization Based on Deep Graph Search,” X. Wu et al. developed an approach which is able to automatically retrieve disease related knowledge in a summarized form from the large volume of online biomedical literature. This approach is capable of finding both direct relations between diseases and genes as well as indirect obscure relationships among diseases and other biomedical entities. Their experiment results show that a precision of 0.6 and a recall of 0.61 can be achieved on extracting bladder cancer-related genetic entities compared to a reference standard recorded in the Online Mendelian Inheritance in Man (OMIM) and Genetics Home Reference database.

The large amount of biomedical literature provides useful knowledge resource for researchers to form biomedical hypotheses. In their work entitled “Supervised Learning Based Hypothesis Generation from Biomedical Literature,” S. Sang et al. proposed a supervised learning-based approach to generate hypotheses from biomedical literature. This approach splits the traditional processing of hypothesis generation model into two models, the AB model and the BC model, which are constructed with a supervised learning method. The purpose of the AB model is to determine whether a physiological phenomenon is caused by a disease in a sentence, and the BC model is used to judge whether there exists an entity having physiological effects on human beings in a sentence. The experimental results on the three classic Swanson's hypotheses demonstrate that the proposed approach can achieve better performance in comparison to concept cooccurrence and grammar engineering-based approaches like SemRep.

DNA, RNA, microRNA, genes, and their products play important roles in molecular biology. Recognizing them from published literature resources is a fundamental and important step in linking molecular biology observations to the clinical world. In contrast to previously developed tools, which are typically restricted to identifying genes mentions in the literature, the paper by C.-H. Wei et al. entitled “GNormPlus: An Integrative Approach for Tagging Genes, Gene Families, and Protein Domains” implemented an open source system that is able to detect genes, gene families, and protein domains. Furthermore, a corpus that allows the development of methods for distinguishing gene-related entities was created in this work. The new system, GNormPlus, achieved an *F*1-score of 0.867 on the BioCreative II gene normalization task dataset. This tool may be used to potentially solidify the automatic recognition of gene-related biological entities buried in the literature and transform the information into valuable resources.

Due to the lack of function and resource requirement in the existed RNA-Seq databases, the study “MetaRNA-Seq: An Interactive Tool to Browse and Annotate Metadata from RNA-Seq Studies” presented by P. Kumar et al. constructed a web service to browse, search, and annotate RNA-Seq metadata for Homo sapiens interactively at the study level. This web tool provides enhanced utilization of metadata for RNA-Seq studies in NCBI resources with semiautomatic curation, structural presentation, and a convenient browser for study- or project-level metadata. The MetaRNA-Seq web service provides a semiautomatic curation pipeline and a friendly interface for annotating metadata. Reviewing the annotated studies with the guided search function provided by MetaRNA-Seq is indeed a supplement to the RNA-Seq metadata resources.

Clinical studies focus on targets including diseases, symptoms, drugs, patients, laboratory measurements, and images within clinical data such as electronic health records. However, previous study has revealed that approximately half of the electronic health record data is stored as free text. Unstructured format as such makes it difficult to retrieve meaningful information. The contribution by H.-J. Dai et al. “Recognition and Evaluation of Clinical Section Headings in Clinical Documents Using Token-Based Formulation with Conditional Random Fields” proposed a token-based formulation with the conditional random field model to establish an electronic health record section heading recognition system. The proposed method provides an integrated solution without the requirement to develop additional heuristics rules for isolating the heading from the surrounding section contents. The authors discussed the existing approaches in section heading recognition and the possibility of applying the method to enhance the meaningful use of electronic health records. They compiled a section heading recognition corpus on the i2b2 2014 clinical dataset and used it to evaluate the performance of the developed system. The proposed method achieved a satisfactory *F*-score of 0.942, which outperformed the sentence-based approach and the dictionary-based system.

Heart disease is the leading cause of death worldwide. The study by J. Jonnagaddala et al. entitled “Identification and Progression of Heart Disease Risk Factors in Diabetic Patients from Longitudinal Electronic Health Records” devised methods to extract heart disease risk factor information from unstructured electronic health records and track their progression. The study has identified the extraction of lab values and test results from unstructured records as a challenging task. The authors employed a hybrid approach combining both rule-based and machine learning clinical text mining techniques and achieved an averaged overall micro *F*-score of 0.8302 for identifying and tracking risk factors including coronary artery diseases, diabetes, hyperlipidemia, hypertension, medication, obesity, family illness, and smoking histories. Nevertheless, their approach obtained unsatisfactory results in extracting information of coronary artery disease and related medications.

Through this special issue, we hope to shed light on the current major advancements in the area of text mining for translational bioinformatics and seize the attention of the scientific community to pursue further investigation and propel more potential applications of text mining techniques in translational bioinformatics research.

